# Vascular Diseases and Metabolic Disorders

**DOI:** 10.1155/2016/5810358

**Published:** 2016-10-19

**Authors:** Ying-Mei Feng, Catherine Verfaillie, Hong Yu

**Affiliations:** ^1^Stem Cell Institute, Leuven University, 3000 Leuven, Belgium; ^2^Beijing Key Laboratory of Diabetes Prevention and Research, Lu He Hospital, Capital Medical University, Beijing 101149, China; ^3^Zhejiang University, Hangzhou 310027, China

 Vascular diseases include cardiovascular and peripheral vascular diseases. For decades, cardiovascular diseases (CVD) stay the number one mortality worldwide. In more details, coronary heart disease or stroke alone caused around 1 of every 6 deaths or 1 of every 19 deaths in the United States in 2010, respectively [1]. The total direct and indirect cost of CVD remains higher than any other diagnostic groups such as cancer [1].

In contrast to CVD, peripheral vascular diseases suffer lack of attention because most of the affected individuals are asymptomatic. The prevalence of peripheral vascular diseases is increasing which reduces the life quality and exposes the risk of infection and thrombosis. Atherosclerosis serves as the common pathogenesis of peripheral arterial disease and coronary heart disease. Therefore, both types of diseases share the same risk factors. For instance, a recent study demonstrated reduced number of endothelial progenitor cells in patients with CVD [2] and PAD [3].

Patients with vascular diseases are always featured as raised blood pressure, obesity, diabetes, and dyslipidemia, all of which constitute metabolic syndrome. From 2003-2004 to 2011-2012, the prevalence of the metabolic syndrome increased from 32.9% to 34.7% [4]. When compared to healthy controls, cardiovascular mortality was 1.6-fold higher in the subjects who had metabolic syndrome [5]. Up to date,* in vitro* and animal studies have consistently illustrated that metabolic disorders disrupt endothelium integrity, promote inflammation and thrombosis, and thus accelerate the progression of vascular diseases [6–8].

Physically, damaged endothelial cells and cardiomyocytes could be replaced by proliferation of neighboring resident cells or stem/progenitor-mediated repair. However, in the occurrence of vascular diseases and metabolic disorders, the balance between cell damage and repair is twisted. Because of its fundamental potential in self-renewal and multilineage differentiation capacity, stem cell-related therapy has developed and reformed the manner of remodeling human degenerative diseases, which could be applied for diagnosis, drug screening, and the likelihood for therapy. Among all types of stem cells, mesenchymal stem cells MSCs are one of the most promising ones for translational application. A number of preclinical studies have employed MSC for the treatment of cardiomyopathy, vascular stenosis, and corneal disease [9].

In the special issue, studies from clinical and basic research were selected that presented the current status of vascular diseases and metabolic disorders. Clinical results brought updated findings on Acute Coronary Syndrome as well as peripheral artery disease, aortic aneurysms, and diabetic microvascular complications. We were informed about the effect of stem cell therapy in the treatment of vascular diseases. The report of “type 2 diabetes mellitus susceptible to pulmonary tuberculosis” enriched our knowledge on diabetes. From the basic research aspect, we got to know more about the physiology of endothelial cells and brown adipocytes.

*Ying-Mei Feng* *Ying-Mei Feng* *Catherine Verfaillie* *Catherine Verfaillie* *Hong Yu* *Hong Yu*
